# Healthful Plant-Based Dietary Patterns Associated with Reduced Adverse Effects of Air Pollution on COPD: Findings from a Large Cohort Study

**DOI:** 10.3390/nu17061055

**Published:** 2025-03-17

**Authors:** Tianrun Wang, Chenyu Zhao, Xiaoqi Fang, Jia Zhao, Wangzhe Chao, Yacong Bo, Liting Zhou

**Affiliations:** 1 School of Public Health, Jilin University, Changchun 130022, China; 15617621703@163.com (T.W.); 17861528838@163.com (X.F.); 18304448578@163.com (J.Z.); chaowz2721@mails.jlu.edu.cn (W.C.); 2 College of Public Health, Zhengzhou University, Zhengzhou 450001, China; zcy8715@gs.zzu.edu.cn

**Keywords:** plant-based diet, chronic obstructive pulmonary disease, air pollution, cohort study, combined effects

## Abstract

Objectives: The potential of a plant-based diet (PD) to mitigate the adverse effects of long-term air pollution exposure on chronic obstructive pulmonary disease (COPD) remains uncertain. This study aims to explore both the independent and synergistic impacts of air pollution components and PD on COPD risk. Methods: Annual concentrations of air pollutants, including particulate matter (PM_2.5_, PM_2.5–10_, and PM_10_), as well as nitrogen oxides (NO_X_) and nitrogen dioxide (NO_2_), were estimated using a land-use regression model. We calculated the plant-based diet index (PDI), healthy plant-based diet index (hPDI), and unhealthy plant-based diet index (uPDI) by evaluating scores of 17 food categories. Cox regression was performed to evaluate their individual and combined effects on COPD risk. Results: This prospective cohort study included 162,741 participants. Every standard deviation increase in PM_2.5_, NO_2_, and NO_X_ exposure was associated with an increased risk of COPD, with an adjusted HR (95% CI) of 1.049 (1.019, 1.079), 1.065 (1.034, 1.096), and 1.063 (1.035, 1.092), respectively. Compared with low-quality hPDI, moderate- and high-quality hPDI were associated with a lower risk of COPD with an adjusted HR (95% CI) of 0.884 (0.827, 0.946) and 0.758 (0.697, 0.825), respectively. For the combined effects, with the level of hPDI increasing, the joint effects of hPDI with PM_2.5_, NO_2_, and NO_X_ showed a gradually increasing negative impact on COPD risk (*p*-interaction = 0.001, 0.005, and 0.005, respectively). Conclusions: Exposure to PM_2.5_, NO_2_, and NO_x_ may elevate the risk of chronic obstructive pulmonary disease (COPD), whereas adherence to a high-quality hPDI could potentially counteract this association. Future research should explore the underlying biological mechanisms, assess the long-term effects of diet, and evaluate the effectiveness of dietary modifications in diverse populations to inform targeted prevention policies.

## 1. Introduction

A major worldwide issue, chronic obstructive pulmonary disease (COPD) has a substantial impact on economic and public health systems [1]. Data from the Global Burden of Disease (GBD) study indicate that COPD contributes to the death of at least 2.9 million people annually, potentially accounting for 7.8% of all deaths in 2023 [2]. Air pollution stands as a leading risk factor for mortality related to respiratory conditions, with emerging evidence highlighting its impact on COPD [3,4,5]. Chronic inflammation, oxidative stress, and autoimmunity, as the key pathogenic mechanisms of COPD [6], can be induced by air pollution, further inducing the adverse effects on the respiratory system and potentially contributing to the development of COPD.

Previous research has demonstrated that dietary habits play a significant role in both the development and progression of COPD. Specifically, the consumption of processed meat has been associated with an increased risk of COPD, particularly among middle-aged women [7]. Moreover, a Western dietary pattern—defined by high consumption of processed meats and low intake of fruits, vegetables, and dietary fiber—has been linked to a higher risk of COPD [8]. In contrast, a Mediterranean diet (MED), rich in fruits, vegetables, oily fish, and whole grains, has been associated with preserved lung function [9]. Recently, the health effects of a dietary pattern based on a plant-based diet (PD) have attracted increasing attention. PD, which involves a higher consumption of plant-derived foods while limiting or excluding animal products, has gained popularity for its health benefits in managing conditions such as diabetes, cognitive impairment, and hypertension [10]. Mounting evidence indicates that PD may play a beneficial role in the prevention of numerous chronic diseases [11,12]. One study revealed that a healthful PD was linked to a significantly reduced risk of developing chronic obstructive pulmonary disease (COPD) [13]. However, the dietary pattern in this study was evaluated based on the frequency of food intake rather than the quantity of food consumed.

Air pollution is linked to a broad spectrum of health issues. Exposure to particulate matter has been connected to detrimental health outcomes, including reduced lung function, respiratory symptoms, cardiovascular disease, and premature mortality [14]. Furthermore, air pollution plays a significant role in the pathogenesis of COPD on a global scale. Recent estimates indicate that approximately 50% of the total attributable risk of COPD may be tied to air pollution [15]. Additionally, emerging research has highlighted significant interactions between PD and air pollution. For instance, a study from the UK Biobank revealed that various healthy diets, such as the MED diet and the dietary approaches to stop hypertension (DASH) diet, may mitigate the risk of type 2 diabetes associated with prolonged exposure to nitrogen dioxide (NO_2_) and nitrogen oxides (NO_x_) [16]. Nevertheless, the combined effects of these dietary patterns and air pollution on COPD remain poorly understood.

In this study, we calculated a plant-based diet index (PDI) based on the UK Biobank cohort to evaluate participants’ PD. We also explored the prospective associations between PD and air pollution with COPD separately. Finally, we integrated PD and air pollution exposure to explore their prospective associations with COPD and evaluated their additive interactions. This study aims to investigate both the independent and combined effects of air pollution components and PD, with a specific focus on healthful PD, in relation to COPD risk.

## 2. Materials and Methods

### 2.1. Study Participants

The data were sourced from the UK Biobank, a pioneering initiative that has amassed an unparalleled wealth of biological and medical information from half a million participants since 2006 [17]. We included 502,371 participants who completed the baseline questionnaire. Participants who withdrew consent during follow-up, had completed fewer than one 24 h dietary assessment, had no data on air pollution exposure, or had COPD at baseline were excluded. In the final analysis, 162,741 participants were included (Figure S1). The UK Biobank study received ethical approval from the National Health Service North-West Multi-Centre Research Ethics Committee, and all participants provided written informed consent of their participation.

### 2.2. Basic Information of the Subject

Baseline data included age, sex, ethnicity, educational level, household income, body mass index (BMI), and smoking and drinking status.

### 2.3. Dietary Assessment and Calculation of Plant-Based Diet Indices

Dietary data were collected using a 24 h dietary recall questionnaire [18]. We calculated PDI, healthful plant-based diet (hPDI), and unhealthful plant-based diet index (uPDI) by evaluating the scores of 17 food categories following a previously used approach [19].

For PDI, healthy plant foods (whole grains, fruits, vegetables, nuts, legumes and vegetarian protein alternatives, tea, and coffee) and unhealthy plant foods (fruit juices, refined grains, potatoes, sugar-sweetened beverages, sweets, and desserts) received positive scores, while animal foods (animal fats, dairy, eggs, fish/seafood, poultry/red meat, miscellaneous animal-based foods) received reverse scores. For hPDI, healthy plant foods received positive scores, while unhealthy plant foods and animal foods received reverse scores. For uPDI, unhealthy plant foods received positive scores, while healthy plant foods and animal foods received reverse scores.

The 17 food groups with an intake of over 0 portions were ranked into quartiles. With positive scores, participants received a score of 1 to 5 according to their intake portions; an intake of 0 portions received a score of 1, and scores of 2 to 5 were given to each quartile from the lowest to the highest. With reverse scores, the participants received scores of 5 to 1 according to their intake portions, while an intake of 0 portions received a score of 5, and scores of 1 to 4 were given to each quartile from the highest to the lowest. PDI, hPDI, and uPDI scores for each participant were calculated by summing the scores of the 17 food groups. Then, the PDI, hPDI, and uPDI were categorized into three grades (low, <P25; medium, P25–P75; high, ≥P75).

### 2.4. Air Pollution Estimates

Air pollutants, including particulate matter (PM) with diameters of <2.5 μm (PM_2.5_), <10 μm (PM_10_), and between 2.5 and 10 μm (PM_2.5–10_), as well as nitrogen dioxide (NO_2_) and nitrogen oxides (NO_x_), were assessed using a land-use regression model. This model was developed as part of the European Study of Cohorts for Air Pollution Effects program to estimate outdoor pollutant concentrations [20,21]. Individual exposure levels were determined based on the annual average concentrations of these pollutants in 2010. Then, air pollutants were categorized into three grades (low, <P25; medium, P25–P75; high, ≥P75).

### 2.5. Ascertainment of COPD

The primary health outcome of this study was COPD, defined according to the International Classification of Diseases, 10th Revision (ICD-10) codes: J40, J41, J42, J43, and J44 [22,23]. COPD diagnoses were identified through hospital inpatient records and death registries, with participants classified as having COPD if they developed the condition during the follow-up period.

The Scottish Morbidity Record, the Patient Episode Database for Wales, and the Hospital Episode Statistics for England were linked to acquire data on hospital admissions and diagnoses. Mortality data, including the date of death, were retrieved from death certificates provided by the National Health Service Information Centre for England and Wales and the National Health Service Central Register for Scotland. Hospital admission records were available until 31 October 2022 for England, 31 August 2022 for Scotland, and 31 May 2022 for Wales, while death records were accessible until 30 November 2022. The earliest death, loss to follow-up, or the last date of available health data was used to censor follow-up.

### 2.6. Missing Data

Multiple imputation by chained equations was used to create a complete dataset with five imputations in order to manage missing covariate data. To guarantee reliable statistical inference, model coefficients were first computed independently inside each imputed dataset and then combined using Rubin’s methods.

### 2.7. Statistical Analysis

Statistical analyses were performed using SAS (version 9.4). Continuous variables were summarized as means with SDs or medians with IQRs, while categorical variables were expressed as numbers with percentages. Differences in categorical variables were assessed using the chi-square test. Cox proportional hazard regression models were employed to examine the individual and combined effects of air pollution and PD on the risk of COPD. The proportional hazard assumption was tested using Schoenfeld residuals, with no violations detected. Two models were constructed: Model 1 was unadjusted, while Model 2 was adjusted for age, sex, ethnicity, educational level, household income, BMI, smoking status, and alcohol consumption. Kaplan–Meier curves, accompanied by log-rank tests, were generated to illustrate the cumulative incidence rates across different categories of dietary patterns or air pollution exposure among all participants. A multiplicative interaction term, ‘air pollution × PD’, was incorporated into Model 2 to explore potential multiplicative interaction effects. Additionally, relative excess risks due to interaction (RERIs) were calculated to evaluate additive interactions. Further details on the calculation methods can be found in prior studies [24,25]. In brief, we coded the risk factors as reference groups: a low-pollution category and a high hPDI category. The Delta method was used to calculate the confidence interval (CI) for the RERI. If the CIs of the RERIs include 0, it means there is no additive interaction.

Subgroup analyses were conducted separately, stratifying by categories of air pollution exposure and PD to investigate their associations. The participants were divided into nine groups for the analysis of combined effects, using the group with a high level of adherence to PD and low air pollution exposure as the reference.

To ensure the robustness of the findings, a series of sensitivity analyses were performed. Initially, we excluded participants who developed COPD within 2 years of follow-up. Second, participants with emphysema at baseline were excluded. Then, individuals with missing covariates were excluded. In addition, we further adjusted for family history of respiratory disease, physical activity, and occupational exposure to dust and fumes, respectively, based on Model 2. Finally, the combined effects of air pollution and PD on COPD were explored in urban and rural areas separately to reveal additional insights.

The significance level for all two-sided tests was set at *p* < 0.05.

## 3. Results

### 3.1. Characteristics of Participants

The distribution of baseline characteristics of the participants is shown in Table 1. The cohort included 162,741 participants from the UK Biobank. During a median follow-up of 13.3 years, 4680 cases of COPD were identified, with an incidence rate of 2.9%. Compared to participants without COPD, those with COPD were older, male, smokers, with lower educational levels and household incomes and higher BMI levels.

### 3.2. Characteristic Distribution of Air Pollution and Plant-Based Dietary Pattern

Table S1 presents the distribution of air pollutants. The mean (SD) concentrations were 9.92 (1.04) μg/m^3^ for PM_2.5_, 6.41 (0.89) μg/m^3^ for PM_2.5–10_, and 16.18 (1.91) μg/m^3^ for PM_10_, with corresponding medians (P25, P75) of 9.86 (9.22, 10.49), 6.11 (5.84, 6.62), and 16.02 (15.20, 16.99) μg/m^3^, respectively. For gaseous pollutants, the mean (SD) concentrations of NO_2_ and NO_x_ were 26.26 (7.74) μg/m^3^ and 43.06 (15.50) μg/m^3^, with medians of 25.64 (20.81, 31.02) μg/m^3^ and 41.33 (33.28, 49.89) μg/m^3^, respectively. These consistent exposure levels, indicated by relatively narrow interquartile ranges, suggest that the study population experienced a stable level of air pollutant exposure.

Similarly, Table S2 details the distribution of PD scores. The PDI had a mean (SD) of 52.62 (5.92) and a median (P25, P75) of 53.00 (49.00, 57.00). The hPDI showed a slightly higher mean (SD) of 56.10 (6.74) and a median of 56.00 (52.00, 61.00), whereas the uPDI had a mean (SD) of 54.14 (6.67) and a median of 54.00 (50.00, 59.00). The data reveal moderate variability in dietary patterns, with a trend toward eating a healthier PD in a substantial segment of the population.

### 3.3. Associations Between COPD Risk and Air Pollution Exposure or Plant-Based Dietary Pattern

The main effects of air pollution exposure and PD are shown in Table 2. An increase in one standard deviation (SD) in exposure to PM_2.5_, NO_2_, and NO_x_ was associated with an elevated risk of COPD incidence. The adjusted hazard ratios (HRs) and their 95% confidence intervals (CIs) were as follows: 1.049 (1.019, 1.079) for PM_2.5_, 1.065 (1.034, 1.096) for NO_2_, and 1.063 (1.035, 1.092) for NO_x_. In contrast, a one-SD increase in PM_2.5–10_ and PM_10_ showed no significant association with COPD risk, with adjusted HRs (95% CIs) of 1.005 (0.977, 1.034) and 1.014 (0.985, 1.044), respectively. Compared with the low-PM_2.5_ group, the moderate- and high-PM_2.5_ groups had HRs of 1.043 (0.969, 1.123) and 1.097 (1.009, 1.192), respectively, after adjusting for confounding factors. Similarly, compared with the low-PM_2.5–10_ group, the moderate- and high-PM_2.5–10_ groups had HRs of 1.103 (1.026, 1.186) and 1.087 (1.000, 1.181), respectively. In comparison with the low-PM_10_ group, the moderate- and high-PM_10_ groups had HRs of 1.017 (0.946, 1.092) and 1.042 (0.960, 1.131), respectively. Furthermore, compared with the low-NO_2_ group, the moderate- and high-NO_2_ groups exhibited HRs of 1.109 (1.030, 1.194) and 1.182 (1.086, 1.287), respectively. The moderate- and high-NOx groups, compared with the low-NOx group, had HRs of 1.070 (0.994, 1.152) and 1.129 (1.038, 1.227), respectively.

Compared with the low-PDI group, the moderate- and high-PDI groups demonstrated HRs of 0.917 (0.856, 0.981) and 0.891 (0.821, 0.966), respectively. For the low-hPDI group, the moderate- and high-hPDI groups had HRs of 0.884 (0.827, 0.946) and 0.758 (0.697, 0.825), respectively. Moreover, compared with the low-uPDI group, the moderate- and high-uPDI groups had HRs of 1.100 (1.023, 1.183) and 1.235 (1.139, 1.340), respectively. Notably, decreasing trends were observed in the associations between COPD and the categories of PDI and hPDI, while increasing trends were observed in the associations between COPD and the categories of PM_2.5_, NO_2_, NO_x_, and uPDI.

The cumulative incidence was higher for participants with low PDI, low hPDI, high uPDI, and high air pollution level, according to cumulative risk analyses we performed to further validate their link over follow-up time (Figures S2 and S3).

### 3.4. Combined Effects of Air Pollution Exposure and Plant-Based Dietary Pattern on COPD

We examined the multiplicative interaction of five air pollution components with three PD scores. The results showed significant interactions between hPDI and PM_2.5_ (*p*-interaction = 0.001), NO_2_ (*p*-interaction = 0.005), and NO_X_ (*p*-interaction = 0.005) in model 2, respectively. We then explored the additive interactions and combined effects of hPDI with PM_2.5_, NO_2_ and NO_X_, respectively (Table 3).

On an additive scale, positive interactions were observed between hPDI categories and PM_2.5_, NO_2_, and NO_X_ exposure on the incidence of COPD. For individuals with low hPDI and high PM_2.5_, medium hPDI and high PM_2.5_, low hPDI and high NO_2_, low hPDI and high NO_X_, and medium hPDI and high NO_X_, the RERIs (95% CI) were 0.44 (0.21, 0.67), 0.26 (0.07, 0.45), 0.48 (0.21, 0.75), 0.41 (0.17, 0.66), and 0.24 (0.04, 0.44) in model 2, respectively.

**Figure 1:**
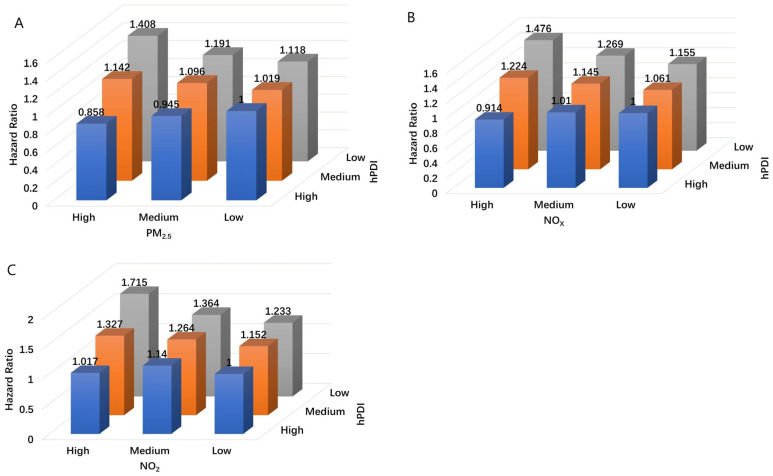
Combined effects of hPDI and PM_2.5_ (**A**)/NO_2_ (**B**)/NO_X_ (**C**) with COPD. Model was adjusted for age, sex, ethnicity, educational level, household income, BMI, and smoking and drinking status.

The combined effects of hPDI and exposure to PM_2.5_, NO_2_, and NO_x_ on COPD are shown in Table 3 and Figure 1. Participants with low hPDI and high NO_2_ exposure were associated with the highest risk of developing COPD. Compared with participants with high hPDI and low NO_2_ exposure, those with low hPDI and high NO_2_ exposure had an HR of 2.117 (1.793, 2.500) in model 1 and 1.715 (1.450, 2.028) in model 2. When compared to participants with high hPDI and low PM_2.5_ or NOx exposure, those with low hPDI and high PM_2.5_ (model 1 HR: 1.869, 95% CI: 1.596, 2.188; model 2 HR: 1.408, 95% CI: 1.200, 1.652) or high NO_x_ (model 1 HR: 1.885, 95% CI: 1.604, 2.216; model 2 HR: 1.476, 95% CI: 1.253, 1.739) exposure were associated with the highest risk of COPD.

### 3.5. Subgroup Analysis

Figure S4 illustrates the results of the subgroup analyses examining the combined effects of air pollution (PM_2.5_, NO_2_, and NO_x_) and hPDI on COPD risk. Among individuals in the highest PM_2.5_, NO_2_, and NO_x_ exposure category, those with a high hPDI had notably lower HRs: 0.590 (0.502, 0.693) for PM_2.5_, 0.584 (0.496, 0.687) for NO_2_, and 0.611 (0.520, 0.720) for NO_x_ than those with a low hPDI, indicating a potential buffering effect of a healthier PD. At low hPDI levels, those with high air pollution exposure had notably higher HRs: 1.288 (1.106, 1.500) for PM_2.5_, 1.422 (1.218, 1.662) for NO_2_, and 1.304 (1.117, 1.523) for NO_x_ than those with low air pollution exposure.

### 3.6. Sensitive Analysis

To evaluate the robustness of the findings, we performed two additional sensitivity analyses. In both cases, the hazard ratios for COPD risk associated with varying levels of air pollution (PM_2.5_, NO_2_, and NO_x_) and hPDI remained generally consistent with the primary analysis. For instance, even after excluding COPD cases from the previous two years, those with low hPDI and high air pollution exposure had notably higher HRs: 1.421 (1.205, 1.677) for PM_2.5_, 1.704 (1.432, 2.027) for NO_2_, and 1.477 (1.247, 1.750) for NO_x_, compared with participants with high hPDI and low air pollution exposure (Table S3). Among participants without emphysema at baseline, those with a low hPDI and high exposure to air pollution had the highest risk of COPD: 1.458 (1.179, 1.803) for PM_2.5_, 1.751 (1.395, 2.197) for NO_2_, and 1.575 (1.265, 1.960) for NO_x_ (Table S4). Similarly, when participants with missing covariates were excluded, those with low hPDI and high air pollution exposure had notably higher HRs: 1.401 (1.180, 1.664) for PM_2.5_, 1.691 (1.414, 2.022) for NO_2_, and 1.478 (1.240, 1.762) for NO_x_ (Table S5). When the model was further adjusted separately for family history of respiratory disease, physical activity, and occupational exposure to dust and fumes, the group with low hPDI and high air pollution exposure consistently exhibited the highest risk of COPD compared to those with high hPDI and low air pollution exposure (Tables S6–S8). For example, after adjusting for family history of respiratory disease, the HR for high PM_2.5_ exposure combined with low hPDI was 1.415 (1.182, 1.694). Finally, the combined effects of air pollution and PD on COPD risk were examined separately in urban and rural areas (Table S9). For example, in urban settings, individuals with high PM_2.5_ exposure and low hPDI had an HR of 1.292 (1.065, 1.567), whereas in rural areas, there was no significant joint effect of air pollution and hPDI on COPD risk.

## 4. Discussion

In this large prospective cohort study involving 162,741 participants from the UK, we found that exposure to high levels of PM_2.5_, NO_2_, or NO_x_, either individually or in combination, is associated with an increased risk of COPD incidence. We also observed a relationship between higher hPDI levels and lower incidence of COPD, particularly in individuals with higher hPDI levels and lower ambient NO_2_ exposure. As hPDI levels increase, the combined effect of hPDI with PM_2.5_, NO_2_, and NO_x_ was associated with progressively greater protection against COPD risk. Additionally, we identified interactions between hPDI levels and exposure to PM_2.5_, NO_2_, and NO_x_. Our findings suggest that adherence to a healthy PD may help mitigate the harmful effects of PM_2.5_, NO_2_, and NO_x_ exposure.

In line with other research, this study discovered that exposure to PM_2.5_ was linked to a higher chance of developing COPD. Similar studies have also found an association between fine PM exposure and adverse outcomes in COPD [15]. Even though the pathophysiology of pollution-related COPD is still unknown, there is growing evidence that fine PM causes a low-grade inflammatory process in the small airways that is mediated by macrophages and results in bronchiolitis [26]. It is also unclear how other common air pollution components, like NO_2_, relate to patient symptoms or a decline in lung function [27]. However, an increasing number of studies have provided new evidence. A cohort study found positive associations between 35-year exposure to NO_2_ and NO_x_ at residential addresses and the incidence of COPD [28]. A multi-ethnic cohort study found a positive association between NO_x_ (per 50 ppb) and NO_2_ (per 20 ppb) exposure and the risk of COPD [29]. Additionally, a meta-analysis indicated that for every 10 μg/m^3^ increase in NO_2_ exposure, the overall relative risk of COPD increased by 2.0% [30]. We further explored the effect of NO_2_ and NO_X_ on COPD risk. Our results indicate that NO_2_ and NO_X_ were associated with an increase in COPD incidence. Therefore, our research provides more comprehensive and robust evidence for understanding the etiology of COPD.

When included in a balanced diet, some foods and nutrients—particularly nutraceuticals with anti-inflammatory and antioxidant qualities—have been linked to improved pulmonary function and a lower risk of COPD [6]. Following the MED is protective against COPD, according to a nested case–control study [31]. Similarly, a study conducted in a U.S. population revealed that a higher DASH diet score was linked to a reduced risk of COPD [32]. However, research exploring the relationship between PD and COPD risk remains limited. In this study, we assessed PD using data derived from 24 h dietary recalls, categorized into 17 food groups. To better understand the influence of food sources and quality within PD on COPD, we constructed three indices: the overall PDI, the hPDI, and the uPDI. Our findings suggest that both overall and healthful PD are associated with a lower risk of COPD. One potential explanation is that PD, rich in antioxidants, may mitigate oxidative stress and inflammation in the respiratory system, potentially delaying the onset of COPD [33,34]. These results contribute novel insights to the existing body of research.

Although limited evidence currently exists regarding whether the relationship between long-term air pollution exposure and COPD was influenced by PD, emerging studies have suggested that such a dietary pattern may confer benefits for other health outcomes linked to PM_2.5_ exposure. A prospective cohort study spanning 17 years and involving 548,845 participants in the United States discovered that individuals with higher MED scores experienced significantly lower cardiovascular mortality rates in connection with PM_2.5_ and NO_2_ exposures [35]. A study conducted among older individuals in China indicated that PM_2.5_ exposure increased the risk of cognitive decline, but PD significantly modified these associations [36]. In this study, we found that a low healthy PD and high air pollution exposure were associated with a synergistic increase in the risk of developing COPD. Specifically, people with high levels of air pollution should adherence to healthy PD. The primary mechanisms underlying adverse health effects from ambient air pollution exposure were associated with oxidative stress and inflammation [37,38,39]. The abundance of antioxidants and anti-inflammatory nutrients in PD may explain how it modifies the association between air pollution exposure and COPD. Oxidative stress induced by air pollutants can directly harm the airway epithelium and diminish the immune response [40]. Another potential mechanism contributing to the heightened vulnerability of COPD patients to ambient air pollution involves the direct induction of inflammation in the already damaged lungs of COPD patients. This heightened inflammation may further contribute to a reduction in pulmonary function among individuals with COPD [41]. Vegetables and fruits are packed with essential nutrients, such as polyphenols, antioxidant vitamins, and dietary fiber. These compounds are linked to anti-inflammatory and antioxidant effects, which may help reduce inflammation and oxidative stress caused by exposure to environmental pollutants in the respiratory system [42]. Consequently, they could potentially slow the progression of respiratory disorders [43].

Participants with emphysema at baseline, those who developed COPD within two years of follow-up, and those with missing variables were excluded in order to perform sensitivity analyses and evaluate the robustness of our findings. These exclusions did not significantly alter the results, indicating that the associations observed were not driven by early-onset COPD cases or missing data. Further adjustments were made to explore the impact of potential confounders, including family history of respiratory disease, physical activity, and occupational exposure to dust and fumes. The results remained consistent across these models, reinforcing the robustness of our findings.

In this study, the rural population constitutes 15 per cent of the total population. The confidence intervals were wider in rural areas due to the smaller sample size, but the trends remained consistent, suggesting that the detrimental impact of air pollution on COPD risk may be modified by dietary patterns across different environmental settings. Additionally, The Western and Northern European regions have similar climates and natural environments to the UK, and air pollution levels in these regions are likely to be similar to those in the UK. Moreover, dietary habits in Western and Northern Europe, as well as the UK, are primarily characterized by a high intake of meat, potatoes, bread, and dairy products. Therefore, the findings of this study are likely generalizable to residents of Western and Northern Europe. However, for regions that dietary patterns and air pollution levels differ significantly from the UK, caution should be exercised when considering the applicability of these results.

Several potential limitations must be recognized. Firstly, as an observational study, it cannot establish causal relationships, so there is a need to conclusively demonstrate causality in randomized controlled trials in the future. Second, the dietary data were self-reported, which may introduce reporting or recall bias. However, the study participants were recalling dietary information from the past 24 h; therefore, the questionnaire had good reliability and low recall bias. Third, the observational nature of this study introduced the possibility of residual confounding, although we have controlled for as many confounding factors as possible based on the available data. Lastly, the UK Biobank may not fully represent the general population, as it is potentially subject to a “healthy volunteer” selection bias. Additionally, the study participants were predominantly of European descent, which may limit the generalizability of the findings to other racial and ethnic groups.

## 5. Conclusions

A higher chance of developing COPD was linked to exposure to PM_2.5_, NO_2_, and NO_x_. The possible preventive function of a healthy PD was highlighted by the fact that following a high-quality hPDI seemed to reduce this risk. These results highlight the significance of integrating dietary interventions into public health strategies for COPD prevention, particularly in regions experiencing high levels of air pollution. Future research should explore the underlying biological mechanisms, assess long-term dietary effects, and evaluate the effectiveness of dietary modifications in diverse populations to inform targeted prevention policies.

## Figures and Tables

**Table 1:** Baseline characteristics of the included participants.

Variables ^a^	All (*n* = 162,741)	Participants Without COPD (*n* = 158,061, 97.1%)	Participants with COPD (*n* = 4680, 2.9%)	*p*
Age				<0.001
<60 years	96,947 (59.6)	95,441 (60.4)	1506 (32.2)	
≥60 years	65,794 (40.4)	62,620 (39.6)	3174 (67.8)	
Sex				<0.001
Female	89,962 (55.3)	87,892 (55.6)	2070 (44.2)	
Male	72,779 (44.7)	70,169 (44.4)	2610 (55.8)	
Ethnicity				<0.001
White	15,6397 (96.1)	15,1852 (96.1)	4545 (97.1)	
Others	6344 (3.9)	6209 (3.9)	135 (2.9)	
Education level				<0.001
Low	40,005 (24.6)	38,786 (24.5)	1219 (26.0)	
Medium	30,106 (18.5)	29,161 (18.4)	945 (20.2)	
High	80,101 (49.2)	78,560 (49.7)	1541 (32.9)	
None of the above	11,914 (7.3)	10,985 (6.9)	929 (19.9)	
Missing	615 (0.4)	569 (0.4)	46 (1.0)	
Household income				<0.001
<GBP 18,000	21,151 (13.0)	19,814 (12.5)	1337 (28.6)	
GBP 18,000–GBP 30,999	35,398 (21.8)	34,093 (21.6)	1305 (27.9)	
GBP 31,000–GBP 51,999	42,485 (26.1)	41,569 (26.3)	916 (19.6)	
GBP 52,000–GBP 100,000	36,885 (22.7)	36,413 (23.0)	472 (10.1)	
>GBP 100,000	11,009 (6.8)	10,916 (6.9)	93 (2.0)	
Missing	15,813 (9.7)	15,256 (9.7)	557 (11.9)	
BMI				<0.001
<25 kg/m^2^	62,161 (38.2)	60,718 (38.4)	1443 (30.8)	
25–29.9 kg/m^2^	67,096 (41.2)	65,262 (41.3)	1834 (39.2)	
≥30 kg/m^2^	33,029 (20.3)	31,653 (20.0)	1376 (29.4)	
Missing	455 (0.3)	428 (0.3)	27 (0.6)	
Smoking				<0.001
Never	92,029 (56.5)	91,001 (57.6)	1028 (22.0)	
Previous	58,486 (35.9)	56,051 (35.5)	2435 (52.0)	
Current	11,829 (7.3)	10,629 (6.7)	1200 (25.6)	
Missing	397 (0.2)	380 (0.2)	17 (0.4)	
Drinking				<0.001
Never	4800 (2.9)	4656 (2.9)	144 (3.1)	
Previous	4713 (2.9)	4442 (2.8)	271 (5.8)	
Current	153,091 (94.1)	148,832 (94.2)	4259 (91.0)	
Missing	137 (0.1)	131 (0.1)	6 (0.1)	

^a^ n (%) for categorical variables. BMI, body mass index. PDI, plant-based diet index; hPDI, healthful plant-based diet index; uPDI, unhealthful plant-based diet index.

**Table 2:** Associations between COPD risk and air pollution exposure and plant-based dietary pattern.

Model	Low	Medium, HR (95% CI)	High, HR (95% CI)	*p*-Trend ^a^	per SD
PM_2.5_					
Model 1	1	1.150 (1.069, 1.238)	1.257 (1.158, 1.364)	<0.001	1.100 (1.070, 1.131)
Model 2	1	1.043 (0.969, 1.123)	1.097 (1.009, 1.192)	0.028	1.049 (1.019, 1.079)
PM_2.5–10_					
Model 1	1	1.153 (1.072, 1.240)	1.144 (1.053, 1.243)	0.002	1.020 (0.992, 1.049)
Model 2	1	1.103 (1.026, 1.186)	1.087 (1.000, 1.181)	0.059	1.005 (0.977, 1.034)
PM_10_					
Model 1	1	1.089 (1.014, 1.170)	1.131 (1.043, 1.227)	0.003	1.049 (1.020, 1.079)
Model 2	1	1.017 (0.946, 1.092)	1.042 (0.960, 1.131)	0.320	1.014 (0.985, 1.044)
NO_2_					
Model 1	1	1.231 (1.144, 1.325)	1.271 (1.169, 1.381)	<0.001	1.089 (1.059, 1.119)
Model 2	1	1.109 (1.030, 1.194)	1.182 (1.086, 1.287)	<0.001	1.065 (1.034, 1.096)
NO_x_					
Model 1	1	1.193 (1.109, 1.284)	1.259 (1.159, 1.367)	<0.001	1.097 (1.070, 1.125)
Model 2	1	1.070 (0.994, 1.152)	1.129 (1.038, 1.227)	0.005	1.063 (1.035, 1.092)
PDI					
Model 1	1	0.843 (0.788, 0.903)	0.782 (0.722, 0.848)	<0.001	0.906 (0.881, 0.932)
Model 2	1	0.917 (0.856, 0.981)	0.891 (0.821, 0.966)	0.004	0.953 (0.926, 0.981)
hPDI					
Model 1	1	0.821 (0.768, 0.877)	0.647 (0.596, 0.703)	<0.001	0.839 (0.816, 0.863)
Model 2	1	0.884 (0.827, 0.946)	0.758 (0.697, 0.825)	<0.001	0.888 (0.862, 0.915)
uPDI					
Model 1	1	1.054 (0.980, 1.133)	1.189 (1.097, 1.289)	<0.001	1.071 (1.041, 1.102)
Model 2	1	1.100 (1.023, 1.183)	1.235 (1.139, 1.340)	<0.001	1.086 (1.055, 1.118)

Model 1 had no adjustment covariates; Model 2 was adjusted for age, sex, ethnicity, educational level, household income, BMI, and smoking and drinking status. HR, hazard ratio; CI, confidence interval. ^a^ Tests for trends were performed by entering 1, 2, or 3 as a continuous variable in the models for low, medium, and high air pollution.

**Table 3:** Combined effects of hPDI and PM_2.5_/NO_2_/NO_X_ with COPD.

Air Pollution	hPDI	Air Pollution Levels (HR (95% CI))			RERI ^a^		*p* for Interaction ^b^
		High	Medium	Low	High	Medium	
**Model 1**							
PM_2.5_	Low	1.869 (1.596, 2.188)	1.500 (1.295, 1.737)	1.270 (1.069, 1.509)	0.64 (0.38, 0.91)	0.22 (−0.01, 0.45)	<0.001
	Medium	1.389 (1.198, 1.611)	1.290 (1.123, 1.482)	1.084 (0.929, 1.263)	0.35 (0.14, 0.55)	0.19 (0.04, 0.35)	
	High	0.958 (0.802, 1.145)	1.013 (0.867, 1.182)	1			
NO_2_	Low	2.117 (1.793, 2.500)	1.731 (1.486, 2.017)	1.395 (1.167, 1.668)	0.66 (0.36, 0.96)	0.11 (−0.15, 0.37)	0.002
	Medium	1.522 (1.302, 1.780)	1.489 (1.287, 1.722)	1.221 (1.040, 1.432)	0.24 (0.01, 0.47)	0.044 (−0.17, 0.26)	
	High	1.066 (0.887, 1.281)	1.224 (1.043, 1.437)	1			
NO_X_	Low	1.885 (1.604, 2.216)	1.616 (1.394, 1.874)	1.313 (1.103, 1.563)	0.58 (0.30, 0.85)	0.22 (−0.02, 0.46)	0.003
	Medium	1.454 (1.250, 1.691)	1.358 (1.180, 1.563)	1.122 (0.961, 1.311)	0.33 (0.12, 0.55)	0.15 (−0.04, 0.34)	
	High	0.998 (0.834, 1.194)	1.086 (0.929, 1.270)	1			
**Model 2**							
PM_2.5_	Low	1.408 (1.200, 1.652)	1.191 (1.027, 1.381)	1.118 (0.940, 1.329)	0.44 (0.21, 0.67)	0.13 (−0.08, 0.34)	0.001
	Medium	1.142 (0.983, 1.326)	1.096 (0.954, 1.259)	1.019 (0.874, 1.188)	0.26 (0.07, 0.45)	0.13 (−0.05, 0.31)	
	High	0.858 (0.717, 1.026)	0.945 (0.809, 1.104)	1			
NO_2_	Low	1.715 (1.450, 2.028)	1.364 (1.169, 1.592)	1.233 (1.030, 1.475)	0.48 (0.21, 0.75)	0.00 (−0.24, 0.25)	0.005
	Medium	1.327 (1.133, 1.553)	1.264 (1.092, 1.462)	1.152 (0.982, 1.352)	0.15 (−0.07, 0.38)	−0.03 (−0.24, 0.17)	
	High	1.017 (0.846, 1.223)	1.140 (0.971, 1.338)	1			
NO_X_	Low	1.476 (1.253, 1.739)	1.269 (1.093, 1.473)	1.155 (0.970, 1.377)	0.41 (0.17, 0.66)	0.11 (−0.11, 0.33)	0.005
	Medium	1.224 (1.052, 1.425)	1.145 (0.994, 1.318)	1.061 (0.908, 1.240)	0.24 (0.04, 0.44)	0.07 (−0.12, 0.26)	
	High	0.914 (0.764, 1.095)	1.010 (0.863, 1.181)	1			

Model 1 had no adjustment covariates; Model 2 was adjusted for age, sex, ethnicity, educational level, household income, BMI, and smoking and drinking status. HR, hazard ratio; CI, confidence interval; hPDI, healthful plant-based diet index. ^a^ The estimates of RERI were calculated based on the reference group with low air pollution and high hPDI. ^b^ Likelihood tests were applied to test the significance of the interaction term by comparing the model with and without the interaction term.

## Data Availability

The data are available in a public, open access repository. The UK Biobank data are available on application to the UK Biobank: www.ukbiobank.ac.uk/ (accessed on 23 March 2023).

## Supplementary Materials

The following supporting information can be downloaded at https://www.mdpi.com/article/10.3390/nu17061055/s1, Figure S1: Flow chart for inclusion and exclusion of the study subjects; Figure S2: Cumulative COPD incidences for low and high of hPDI (A), PM_2.5_ (B), NO_2_ (C), and NO_X_ (D) in the total population; Figure S3: Cumulative COPD incidences for low and high of PDI (A), uPDI (B), PM_2.5–10_ (C), and PM_10_ (D); Figure S4: Subgroup analyses on the association of COPD with PM_2.5_, NO_2_, and NO_X_ exposure stratified by the category hPDI, and hPDI exposure stratified by the category PM_2.5_, NO_2_, and NO_X_; Table S1: Characteristic distribution of air pollution; Table S2: Characteristic distribution of plant-based dietary pattern; Table S3: Sensitivity analyses 1 (exclusion of participants who developed COPD within 2 years of follow-up); Table S4: Sensitivity analyses 2 (exclusion of participants with emphysema at baseline); Table S5: Sensitivity analyses 3 (exclusion of participants with missing covariates); Table S6: Sensitivity analyses 4 (model further adjusted for family history of respiratory disease); Table S7: Sensitivity analyses 5 (model further adjusted for physical activity); Table S8: Sensitivity analyses 6 (model further adjusted for occupational exposure to dust and fumes); Table S9: Sensitivity analyses 7 (combined effects of hPDI and PM_2.5_/NO_2_/NO_X_ on COPD in urban or rural areas).

## Abbreviations

The following abbreviations are used in this manuscript:

BMI	body mass index
CI	confidence interval
COPD	chronic obstructive pulmonary disease
DASH	dietary approaches to stop hypertension diet
GBD	Global Burden of Disease
hPDI	healthful plant-based diet index
ICD	International Classification of Disease
MED	Mediterranean diet
NO_2_	nitrogen dioxide
NO_x_	nitrogen oxides
PD	plant-based diet
PDI	plant-based diet index
PM	particulate matter
RERI	relative excess risks due to interaction
uPDI	unhealthful plant-based diet index
