# Supplementary material for: Healthful Plant-Based Dietary Patterns Associated with Reduced Adverse Effects of Air Pollution on COPD: Findings from a Large Cohort Study
Source: Nutrients. 2025 Mar 17;17(6):1055. doi: 10.3390/nu17061055 (PMC11946186; doi:10.3390/nu17061055)
Supplement: Supplementary file 1 [file nutrients-17-01055-s001.zip › nutrients-3510456-supplementary.pdf]

1 Supplementary Figures and Tables.

1.1 Supplementary Figures

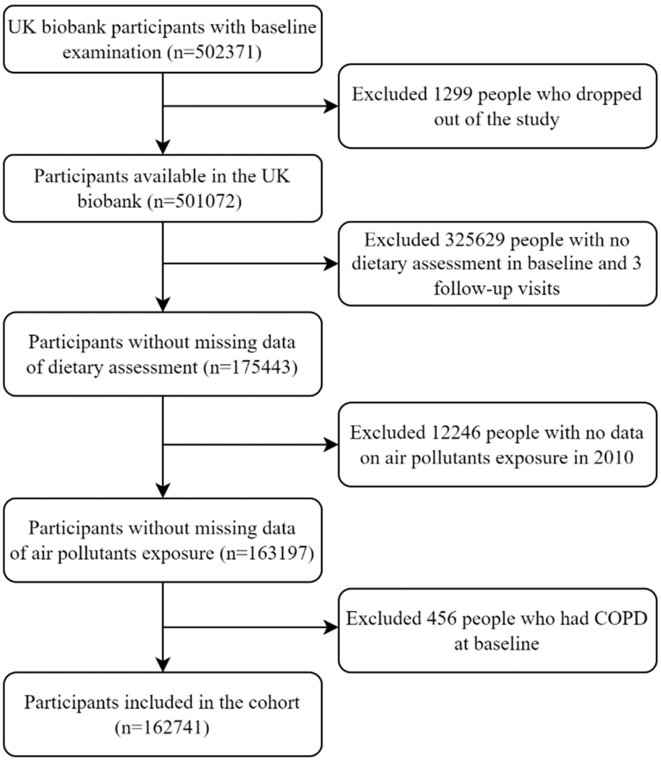

Figure S1. Flow chart for inclusion and exclusion of the study subjects.

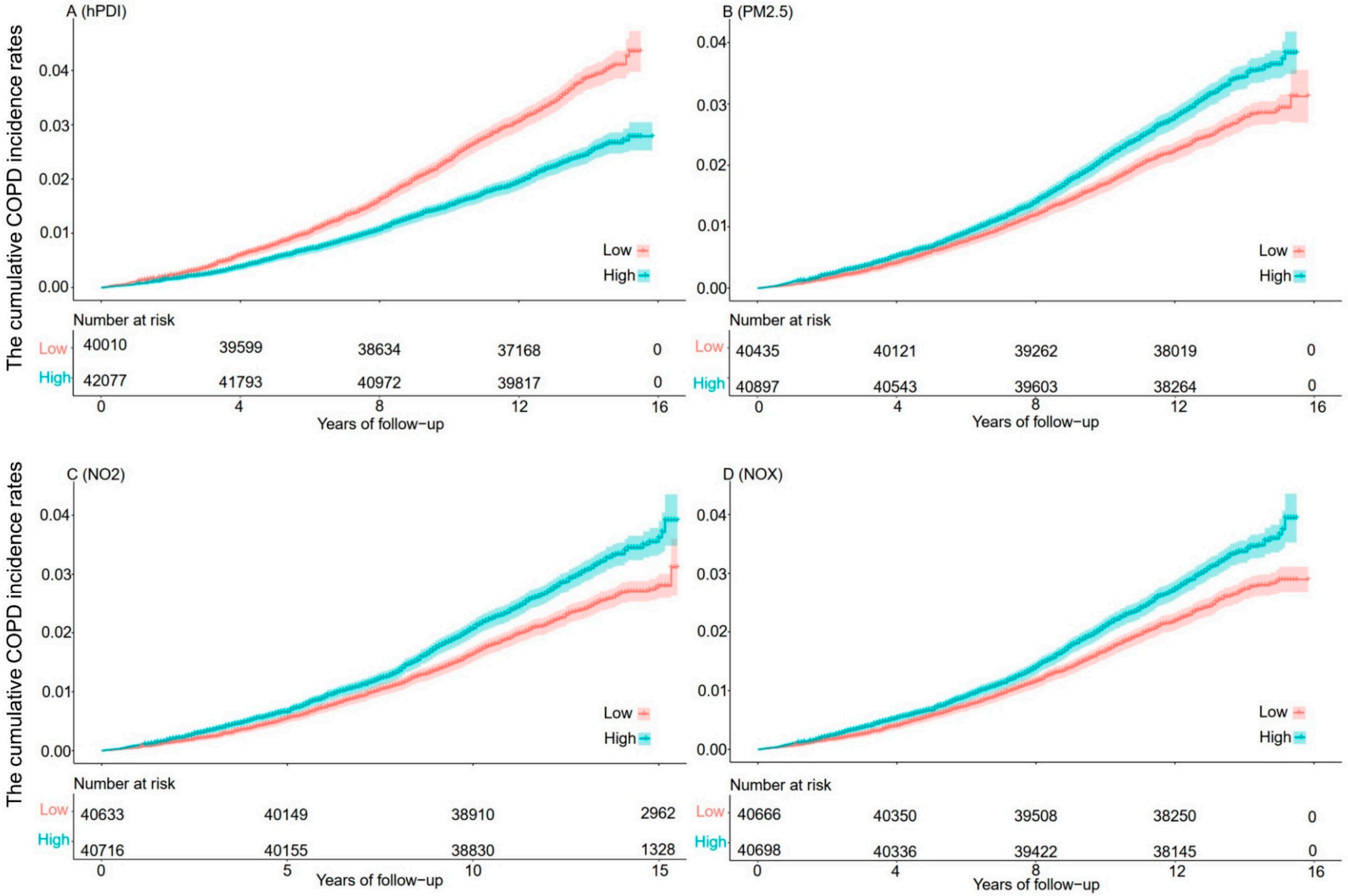

Figure S2. Cumulative COPD incidences for low and high of hPDI (A), $PM_{2.5}$ (B), NO_2 (C) and NO_X (D) in the total population.

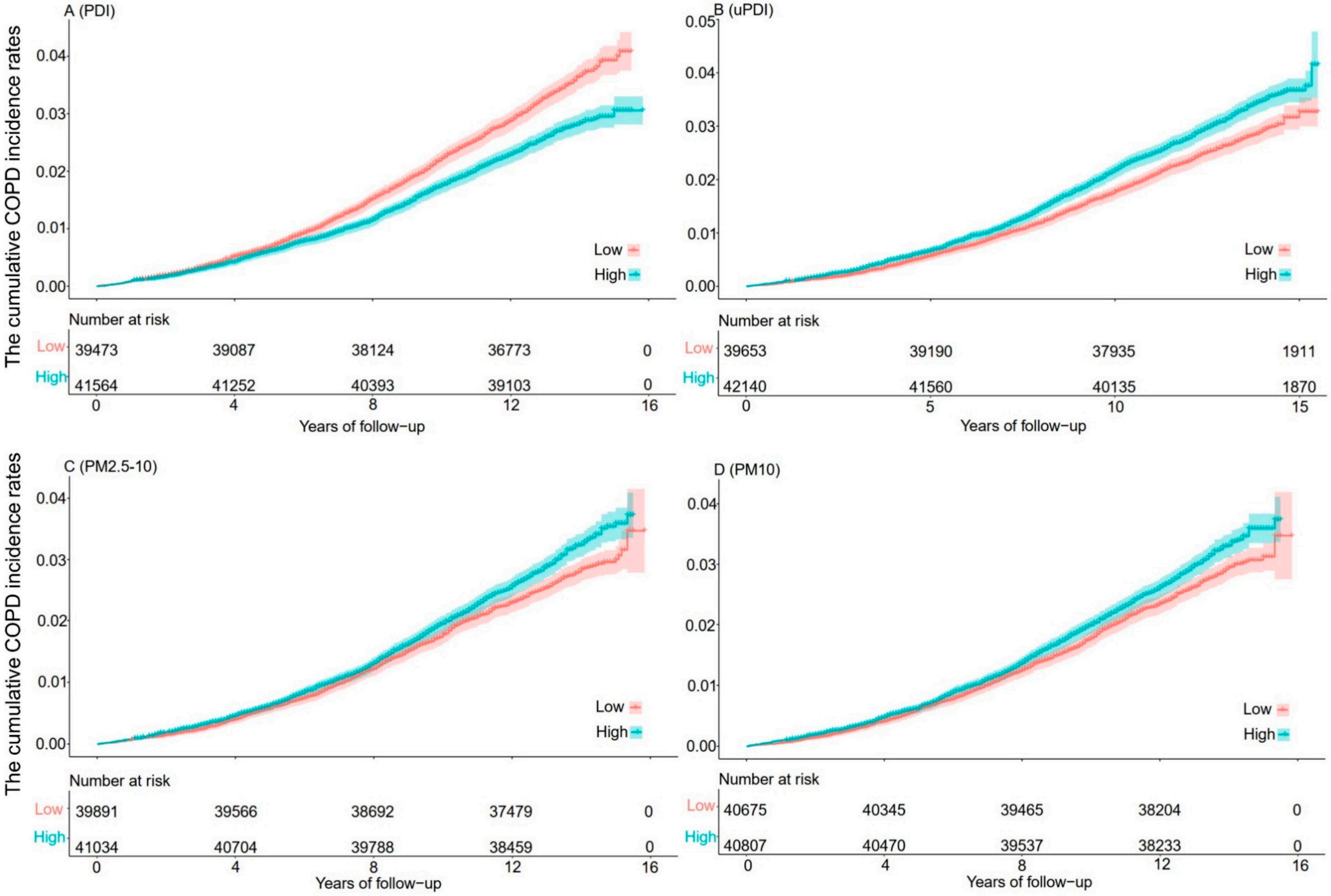

Figure S3. Cumulative COPD incidences for low and high of PDI (A), uPDI (B), $PM_{2.5-10}$ (C) and PM_{10} (D).

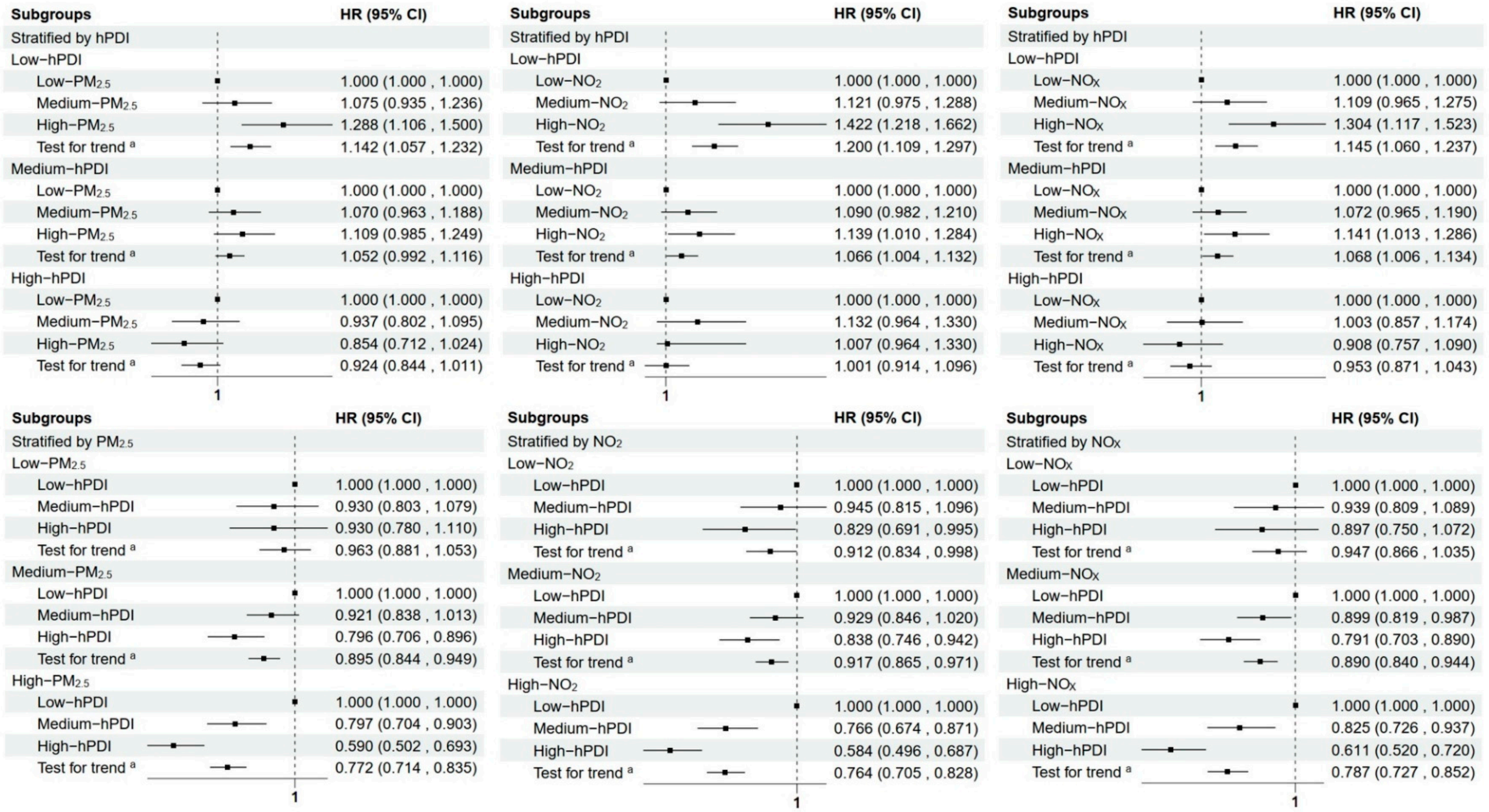

Figure S4. Subgroup analyses on the association of COPD with $PM_{2.5}$, NO_2, and NO_X exposure stratified by the categories of hPDI, and hPDI exposure stratified by the categories of $PM_{2.5}$, NO_2, and NO_X. Model was adjusted for age, sex, ethnic, educational level, household income, BMI, smoking and drinking. [a] Test for trend was performed by entering 1, 2 and 3 as a continuous variable in the models for low,

medium and high of air pollution or plant-based dietary pattern. HR, hazard ratio; CI, confidence interval; hPDI, healthful plant-based diet index.

1.2 Supplementary Tables

Table S1. Characteristic distribution of air pollution.

Components of air pollution	Mean (SD) ($\mu g/m^3$)	Median (P_{25}, P_{75}) ($\mu g/m^3$)
$PM_{2.5}$	9.92 (1.04)	9.86 (9.22, 10.49)
$PM_{2.5-10}$	6.41 (0.89)	6.11 (5.84, 6.62)
PM_{10}	16.18 (1.91)	16.02 (15.20, 16.99)
NO_2	26.26 (7.74)	25.64 (20.81, 31.02)
NO_X	43.06 (15.50)	41.33 (33.28, 49.89)

Table S2. Characteristic distribution of plant-based dietary pattern.

Plant-based dietary pattern	Mean (SD)	Median (P_{25}, P_{75})
PDI	52.62 (5.92)	53.00 (49.00, 57.00)
hPDI	56.10 (6.74)	56.00 (52.00, 61.00)
uPDI	54.14 (6.67)	54.00 (50.00, 59.00)

Table S3. Sensitivity analyses 1 (exclusion of participants who developed COPD within 2 years of follow-up).

Air pollution	hPDI	High-air pollution HR (95%CI)[a]	Medium-air pollution HR (95%CI)[a]	Low-air pollution HR (95%CI)[a]
$PM_{2.5}$	Low	1.421(1.205,1.677)	1.203(1.032,1.402)	1.124(0.939,1.344)
	Medium	1.143(0.980,1.335)	1.110(0.961,1.281)	1.028(0.877,1.205)
	High	0.848(0.704,1.021)	0.947(0.806,1.112)	1
NO_2	Low	1.704(1.432,2.027)	1.375(1.173,1.612)	1.236(1.027,1.487)
	Medium	1.329(1.130,1.563)	1.270(1.092,1.477)	1.151(0.976,1.358)
	High	0.995(0.822,1.205)	1.134(0.961,1.339)	1
NO_X	Low	1.477(1.247,1.750)	1.287(1.103,1.501)	1.155(0.963,1.384)
	Medium	1.221(1.043,1.429)	1.163(1.005,1.346)	1.060(0.902,1.245)
	High	0.908(0.753,1.094)	1.007(0.856,1.184)	1

Model was adjusted for age, sex, ethnic, educational level, household income, BMI, smoking and drinking. HR, hazard ratio; CI, Confidence interval; hPDI, healthful plant-based diet index.

Table S4. Sensitivity analyses 2 (exclusion of participants with emphysema at baseline).

Air pollution	hPDI	High- air pollution HR (95%CI)	Medium- air pollution HR (95%CI)[a]	Low- air pollution HR (95%CI)
$PM_{2.5}$	Low	1.458(1.179,1.803)	1.197(0.981,1.460)	1.148(0.911,1.447)
	Medium	1.270(1.042,1.548)	1.190(0.987,1.435)	1.096(0.892,1.347)
	High	0.942(0.745,1.191)	0.960(0.779,1.183)	1
NO_2	Low	1.751(1.395,2.197)	1.453(1.178,1.793)	1.341(1.053,1.708)
	Medium	1.511(1.223,1.868)	1.438(1.178,1.755)	1.282(1.031,1.595)
	High	1.108(0.866,1.418)	1.244(1.001,1.548)	1
NO_X	Low	1.575(1.265,1.960)	1.302(1.063,1.595)	1.267(1.002,1.602)
	Medium	1.408(1.150,1.725)	1.297(1.071,1.572)	1.142(0.924,1.411)
	High	0.969(0.761,1.233)	1.112(0.900,1.374)	1

Model was adjusted for age, sex, ethnic, educational level, household income, BMI, smoking and drinking. HR, hazard ratio; CI, Confidence interval; hPDI, healthful plant-based diet index.

Table S5. Sensitivity analyses 3 (exclusion of participants with missing covariates).

Air pollution	hPDI	High-air pollution HR (95%CI)[a]	Medium-air pollution HR (95%CI)	Low-air pollution HR (95%CI)[a]
$PM_{2.5}$	Low	1.401(1.180,1.664)	1.212(1.034,1.421)	1.119(0.929,1.348)
	Medium	1.103(0.939,1.296)	1.076(0.926,1.250)	1.040(0.882,1.227)
	High	0.851(0.703,1.031)	0.941(0.796,1.112)	1
NO_2	Low	1.691(1.414,2.022)	1.353(1.148,1.594)	1.210(1.000,1.465)
	Medium	1.270(1.074,1.502)	1.212(1.037,1.417)	1.148(0.968,1.361)
	High	0.960(0.788,1.169)	1.120(0.944,1.329)	1
NO_X	Low	1.478(1.240,1.762)	1.299(1.107,1.525)	1.161(0.962,1.401)
	Medium	1.212(1.030,1.427)	1.126(0.967,1.311)	1.075(0.910,1.271)
	High	0.908(0.749,1.102)	1.017(0.859,1.203)	1

Model was adjusted for age, sex, ethnic, educational level, household income, BMI, smoking and drinking. HR, hazard ratio; CI, Confidence interval; hPDI, healthful plant-based diet index.

Table S6. Sensitivity analyses 4 (Model further adjusted for family history of respiratory disease).

Air pollution	hPDI	High-air pollution HR (95%CI)[a]	Medium-air pollution HR (95%CI)[a]	Low-air pollution HR (95%CI)[a]
$PM_{2.5}$	Low	1.415(1.182,1.694)	1.182(1.000,1.397)	1.102(0.905,1.341)
	Medium	1.104(0.933,1.306)	1.086(0.929,1.270)	1.012(0.851,1.204)
	High	0.827(0.676,1.012)	0.907(0.761,1.081)	1
NO_2	Low	1.721(1.424,2.081)	1.399(1.175,1.664)	1.160(0.943,1.426)
	Medium	1.300(1.088,1.553)	1.265(1.073,1.491)	1.135(0.947,1.361)
	High	0.983(0.798,1.211)	1.110(0.926,1.330)	1
NO_X	Low	1.408(1.168,1.697)	1.354(1.144,1.602)	1.092(0.892,1.335)
	Medium	1.182(0.995,1.405)	1.161(0.990,1.361)	1.068(0.896,1.274)
	High	0.899(0.734,1.102)	0.989(0.828,1.181)	1

Model was adjusted for age, sex, ethnic, educational level, household income, BMI, smoking, drinking and family history of respiratory disease. HR, hazard ratio; CI, Confidence interval; hPDI, healthful plant-based diet index.

Table S7. Sensitivity analyses 5 (Model further adjusted for physical activity).

Air pollution	hPDI	High-air pollution HR (95%CI)[a]	Medium-air pollution HR (95%CI)[a]	Low-air pollution HR (95%CI)[a]
$PM_{2.5}$	Low	1.392(1.167,1.661)	1.157(0.983,1.362)	1.067(0.881,1.294)
	Medium	1.124(0.953,1.325)	1.051(0.902,1.225)	1.028(0.869,1.218)
	High	0.772(0.631,0.945)	0.957(0.808,1.135)	1
NO_2	Low	1.721(1.430,2.072)	1.319(1.112,1.566)	1.235(1.013,1.504)
	Medium	1.297(1.089,1.544)	1.251(1.064,1.471)	1.148(0.962,1.371)
	High	0.967(0.787,1.188)	1.151(0.964,1.374)	1
NO_X	Low	1.473(1.229,1.764)	1.237(1.049,1.460)	1.120(0.923,1.360)
	Medium	1.207(1.020,1.427)	1.120(0.958,1.310)	1.056(0.889,1.254)
	High	0.856(0.700,1.047)	1.019(0.858,1.212)	1

Model was adjusted for age, sex, ethnic, educational level, household income, BMI, smoking, drinking and physical activity. HR, hazard ratio; CI, Confidence interval; hPDI, healthful plant-based diet index.

Table S8. Sensitivity analyses 6 (Model further adjusted for occupational exposure to dust and fumes).

Air pollution	hPDI	High-air pollution HR (95%CI)[a]	Medium-air pollution HR (95%CI)[a]	Low-air pollution HR (95%CI)[a]
$PM_{2.5}$	Low	1.339(1.056,1.699)	1.161(0.934,1.442)	1.111(0.862,1.431)
	Medium	0.982(0.785,1.229)	0.981(0.801,1.203)	0.914(0.729,1.146)
	High	0.729(0.555,0.958)	0.898(0.715,1.128)	1
NO_2	Low	1.748(1.366,2.238)	1.296(1.034,1.624)	1.158(0.889,1.508)
	Medium	1.161(0.918,1.467)	1.104(0.892,1.367)	1.040(0.824,1.313)
	High	0.891(0.674,1.177)	1.061(0.839,1.341)	1
NO_X	Low	1.363(1.069,1.738)	1.233(0.993,1.531)	1.044(0.807,1.350)
	Medium	0.997(0.794,1.251)	0.981(0.799,1.204)	0.977(0.781,1.223)
	High	0.801(0.612,1.049)	0.893(0.710,1.123)	1

Model was adjusted for age, sex, ethnic, educational level, household income, BMI, smoking, drinking and occupational exposure to dust and fumes. HR, hazard ratio; CI, Confidence interval; hPDI, healthful plant-based diet index.

Table S9. Sensitivity analyses 7 (Combined effects of hPDI and $PM_{2.5}$/ NO_2/ NO_X on COPD in urban or rural).

Air pollution	hPDI	High-air pollution HR (95%CI)[a]	Medium-air pollution HR (95%CI)[a]	Low-air pollution HR (95%CI)[a]
**Urban**				
$PM_{2.5}$	Low	1.292(1.065,1.567)	1.077(0.895,1.295)	1.089(0.867,1.368)
	Medium	1.037(0.862,1.247)	1.013(0.849,1.209)	0.917(0.747,1.127)
	High	0.776(0.630,0.957)	0.854(0.705,1.033)	1
NO_2	Low	1.759(1.403,2.206)	1.390(1.120,1.727)	1.318(1.012,1.716)
	Medium	1.367(1.099,1.700)	1.304(1.056,1.609)	1.161(0.913,1.477)
	High	1.034(0.815,1.313)	1.162(0.932,1.450)	1
NO_X	Low	1.434(1.172,1.756)	1.234(1.019,1.495)	1.143(0.902,1.447)
	Medium	1.193(0.984,1.446)	1.108(0.922,1.333)	1.049(0.849,1.297)
	High	0.883(0.712,1.096)	0.976(0.801,1.189)	1

Air pollution	hPDI	High-air pollution HR (95%CI)[a]	Medium-air pollution HR (95%CI)[a]	Low-air pollution HR (95%CI)[a]
**Rural**				
$PM_{2.5}$	Low	1.242(0.455,3.388)	1.444(1.034,2.018)	1.067(0.811,1.404)
	Medium	1.298(0.630,2.674)	0.964(0.712,1.306)	1.146(0.906,1.449)
	High	0.739(0.182,3.004)	0.977(0.652,1.462)	1
NO_2	Low	3.121(0.769,12.666)	1.556(1.058,2.286)	1.072(0.833,1.379)
	Medium	0.310(0.043,2.224)	0.957(0.663,1.381)	1.118(0.900,1.387)
	High	0.894(0.125,6.405)	0.815(0.468,1.419)	1
NO_X	Low	1.727(0.801,3.722)	1.203(0.847,1.708)	1.084(0.829,1.419)
	Medium	0.567(0.231,1.393)	1.144(0.854,1.532)	1.064(0.844,1.342)
	High	0.494(0.122,2.002)	0.880(0.576,1.346)	1

Model was adjusted for age, sex, ethnic, educational level, household income, BMI, smoking and drinking. HR, hazard ratio; CI, Confidence interval; hPDI, healthful plant-based diet index.
